# Replication fork collisions cause pathological chromosomal amplification in cells lacking RecG DNA translocase

**DOI:** 10.1111/j.1365-2958.2009.06909.x

**Published:** 2009-10-26

**Authors:** Christian J Rudolph, Amy L Upton, Robert G Lloyd

**Affiliations:** Institute of Genetics, University of Nottingham, Queen's Medical CentreNottingham, NG7 2UH, UK

## Abstract

Duplication and transmission of chromosomes require precise control of chromosome replication and segregation. Here we present evidence that RecG is a major factor influencing these processes in bacteria. We show that the extensive DnaA-independent stable DNA replication observed without RecG can lead to replication of any area of the chromosome. This replication is further elevated following irradiation with UV light and appears to be perpetuated by secondary events that continue long after the elimination of UV lesions. The resulting pathological cascade is associated with an increased number of replication forks traversing the chromosome, sometimes with extensive regional amplification of the chromosome, and with the accumulation of highly branched DNA intermediates containing few Holliday junctions. We propose that the cascade is triggered by replication fork collisions that generate 3′ single-strand DNA flaps, providing sites for PriA to initiate re-replication of the DNA and thus to generate linear duplexes that provoke recombination, allowing priming of even further replication. Our results shed light on why termination of replication in bacteria is normally limited to a single encounter of two forks and carefully orchestrated within a restricted area, and explain how a system of multiple forks and random termination can operate in eukaryotes.

## Introduction

Duplication and transmission of the genome are major challenges for dividing cells. Studies with numerous model systems have revealed how these challenges are met through interplay between DNA replication, recombination and repair and in eukaryotes, through checkpoint controls that regulate the cell cycle. It requires careful control over the initiation of replication, multiple pathways for rescuing stalled or damaged forks and various means to curb unnecessary recombination (Lopes *et al.*, 2001; McGlynn and Lloyd, 2002; Messer, 2002; Heller and Marians, 2006; Mahdi *et al.*, 2006; Blow and Gillespie, 2008). It must also involve means to terminate DNA synthesis when replication forks meet, although how this last step is achieved is not clear.

Bacteria have evolved systems that tend to bring replication to a halt within a restricted region of the chromosome. In *Escherichia coli,* replication initiates at a single origin (*oriC*) under the control of DnaA protein (Messer, 2002). The two forks established proceed around the circular chromosome in opposite directions until they meet within a broad termination zone opposite the origin. This zone is flanked by polar sequences (*ter*) that allow forks to enter, but not to leave (Mulcair *et al.*, 2006; Duggin *et al.*, 2008). Thus, the chromosome is divided into two replichores and termination is restricted to a specialized area containing additional genetic elements that orchestrate chromosome segregation (Reyes-Lamothe *et al.*, 2008). But the path of the two forks traversing each replichore is not always smooth. If one of the forks becomes blocked and fails to be rescued, the remaining portion of the replichore will not be duplicated in time as the second fork will be trapped in the terminus area (Sharma and Hill, 1995). This constraint imposed by Tus*-ter* may explain why mechanisms promoting fork rescue and replication restart appear to be so important for bacteria (McGlynn and Lloyd, 2002; Heller and Marians, 2006).

In eukaryotes, the multiple origins per chromosome and lack of defined termination sites mean that such incomplete replication would be less likely. Any chromosome segment left un-replicated by a blocked fork could be duplicated by another fork coming from an adjacent origin. Even if converging forks were both blocked, replication could be completed if one of the many dormant origins present in eukaryotic chromosomes was located in between and was induced to fire (Ge *et al.*, 2007). Such new initiation could also provide a solution to blocked forks in bacteria. However, this possibility has received little attention despite evidence that replication can initiate at sites remote from *oriC* and independently of DnaA. This so-called stable DNA replication (SDR) is induced by exposure to genotoxic agents (iSDR) and elevated constitutively (cSDR) in *rnhA* mutants lacking RNase HI (Kogoma, 1997).

Although SDR could in theory obviate the need to rescue a stalled fork, at least in some circumstances, its initiation at various sites in the chromosome would increase the incidence of fork collisions, which may be problematic, as it would interfere with the evolved replichores. Without SDR, fork collisions are thought to be restricted to the terminus area and limited to a single event per cell cycle. Studies of DNA replication *in vitro* revealed that without Tus to curb fork movement, a replisome may displace the 3′ end of the nascent leading strand made by the fork coming in the other direction, generating a branched DNA structure that allows re-replication of the already replicated DNA (Hiasa and Marians, 1994; Krabbe *et al.*, 1997; Markovitz, 2005). Strand displacement is not observed with DnaB alone (Kaplan and O'Donnell, 2002), indicating that re-replication is a particular risk following collision between fully fledged replisomes.

Studies of *E. coli* strains lacking either PriA or RecG protein support the idea that SDR may be a double-edged sword. PriA facilitates loading of the DnaB replicative helicase and subsequent replisome assembly at branched DNAs (Heller and Marians, 2006). It is required for initiating SDR and also for restarting DNA synthesis following exposure to genotoxic agents (Kogoma, 1997; Tanaka *et al.*, 2003; Rudolph *et al.*, 2009). Without PriA, cells show reduced viability and increased sensitivity to genotoxic agents. These phenotypes are generally attributed to a failure to rescue stalled or damaged forks (Heller and Marians, 2006), but the failure to curb unnecessary recombination is another possibility (Mahdi *et al.*, 2006). PriA has a DNA helicase activity that can unwind any 5′ strand at the branch point of a fork to create a landing pad for DnaB (Heller and Marians, 2006). Strains expressing helicase defective PriA show reduced levels of constitutive as well as damage-induced SDR (Tanaka *et al.*, 2003), but lack the severe defects associated with *priA* null strains despite the limited ability of the mutant protein to unwind stalled forks. They also show a reduced requirement for RecG protein to help survive damage to DNA (Al-Deib *et al.*, 1996).

RecG is a dsDNA translocase that has been implicated in the processing of Holliday junction intermediates in recombination and DNA repair, and in the rescue of stalled replication forks (Lloyd, 1991; Lloyd and Sharples, 1993; McGlynn and Lloyd, 2002). However, it has proven difficult to pin down what it does *in vivo.* In a recent study we were able to demonstrate that UV-irradiated *recG* cells show dramatically increased levels of DnaA-independent DNA synthesis and that this is associated with severe and persistent defects in chromosome segregation. Both the segregation defect and the induced synthesis are suppressed in the absence of PriA helicase, opening the possibility that excessive levels of SDR might be responsible for the phenotype of cells lacking RecG (Rudolph *et al.*, 2009). We suggested that UV-irradiation triggers the assembly of new replication forks, thereby increasing the likelihood of unregulated fork collisions, which in the absence of RecG trigger a pathological cascade of replication that interferes with chromosome segregation and delays cell division (Rudolph *et al.*, 2009).

In this paper, we present evidence to support this hypothesis and propose a model to explain how RecG might act to limit such pathological replication by reducing the incidence of unscheduled replication fork collisions, thus maintaining the function of the evolved replichore arrangement of the chromosome. We also consider why the multiple fork collisions intrinsic to chromosome replication in eukaryotes do not appear to trigger a similar pathological cascade.

## Results

### SDR extends DNA synthesis to all chromosomal areas

**Fig. 1:**
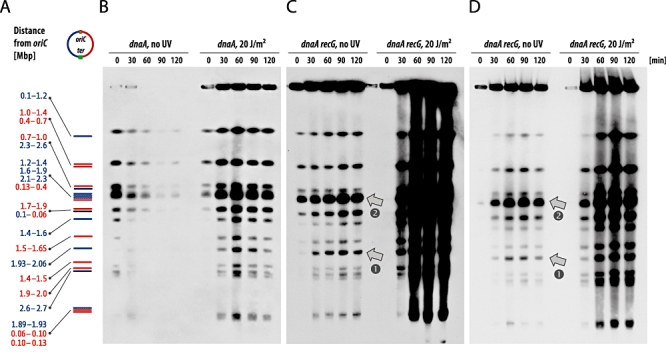
UV-induced *dnaA*-independent replication reaches all chromosomal areas. A. Schematic NotI restriction pattern of the *E. coli* chromosome. The distance from *oriC* to each end of the fragments is indicated. Fragments clockwise and anticlockwise of *oriC* are shown in red and blue, respectively; the fragment containing *oriC* is shown in black. B and C. Visualization of BrdU incorporation in UV-irradiated or mock-irradiated cells in the absence of *oriC* firing. Cells were grown at permissive temperature, UV-irradiated or mock-irradiated and subsequently shifted to 42°C. At the time points indicated the cells were pulse-labelled with BrdU for 10 min. The strains used were AU1054 (*dnaA46*) and AU1091 (*recG dnaA46*). The arrows indicate two fragments that show an increased amount of synthesis in comparison with wild-type cells (compare with Fig. S1B). D. Repeat of the experiment in (C). A reduced exposure time is presented for better visualization of the band pattern of the UV-irradiated sample. For quantification of band intensities see Fig. S1.

To gain further insight into the nature of the SDR induced by UV-irradiation, and that elevated constitutively in cells lacking RecG, we investigated whether it can extend DNA synthesis to any area of the chromosome. We exploited *dnaA46* cells expressing thermosensitive DnaA protein for this purpose. Exponential phase cells were shifted from 30°C to 42°C to eliminate further *oriC* firing and the rate of subsequent DNA synthesis monitored by pulse labelling with BrdU, with or without prior UV-irradiation. High-molecular-weight chromosomal DNA was prepared, digested with the rare cutter NotI and chromosomal fragments separated by pulsed field gel electrophoresis (PFGE) before probing for BrdU (Fig. 1A).

Without irradiation, the rate of DNA synthesis in *dnaA46* cells reduces over time at 42°C in all areas of the chromosome, consistent with synthesis by pre-existing replication forks coming to an end (Fig. 1B). Following irradiation, the pattern changes quite markedly. Initially, the rate of synthesis is reduced (Fig. 1B, time zero), in line with our previous studies showing that all forks stall for 15–20 min (Rudolph *et al.*, 2007). However, the rate then increases substantially and robust labelling occurs in all chromosomal areas for at least another 90 min after irradiation, when the experiment was terminated (Fig. 1B). Thus, robust synthesis continues long after any restarted forks should have reached the terminus. These data confirm our previous results showing that damage-induced SDR is one of the major components contributing towards net DNA synthesis following UV-irradiation (Rudolph *et al.*, 2007). Its persistence is in line with results showing that damage-induced SDR can continue for many hours (Kogoma, 1997).

Robust, DnaA-independent synthesis is detected in unirradiated *recG dnaA46* cells at every time point after the shift to 42°C (Fig. 1C). This is in line with previous results showing that cells lacking RecG show constitutively increased levels of SDR (Hong *et al.*, 1995; Rudolph *et al.*, 2009). In every experiment with unirradiated *recG dnaA46* cells, we observed disproportionately high labelling of two bands (Fig. 1C, white arrows 1 and 2). Such high labelling of these bands is not evident in UV-irradiated *dna46* cells (Fig. 1B), nor in logarithmically growing wild-type cells (Fig. S1B). It may reflect the activity of three reported constitutive SDR origins (*oriK2, oriK4* and *oriK5*) (Kogoma, 1997).

With this exception, SDR-mediated labelling appears to occur simultaneously and proportionately in all areas of the chromosome. It is also detected at all time points sampled following irradiation. In these cases, the level of SDR is dramatically increased, especially in *recG* cells, as reported (Rudolph *et al.*, 2009). Figure 1D shows a second experiment with a lower exposure. It emphasizes the increased labelling of unirradiated cells in the absence of RecG (compare 0 and 30 min lanes in Fig. 1C and D, no UV), and allowed quantification of relative band intensities (Fig. S1). The latter confirmed the disproportionate labelling described above. Overall, these data reveal no particular hotspots for the initiation of SDR following irradiation. Although we cannot exclude the possibility that hotspots do exist (Kogoma, 1997), it seems likely that synthesis can be initiated at any chromosomal locations, at least on a population level. Taken together, the apparently random initiation of SDR and the dramatic increase in synthesis in *dna46 recG* cells over *dnaA46* cells, suggests that UV-irradiated cells lacking RecG may have a much-increased number of forks traversing the chromosome in different directions. If true, this would break the replichore arrangement, thus increasing the danger of fork collisions not only with transcription complexes but also with forks following the normal direction of replication.

### Amplification of SeqA foci in UV-irradiated cells

To try to gain more information about the number of forks traversing the chromosome in *recG* cells following irradiation with UV light, we exploited fluorescently labelled SeqA protein (SeqA-CFP). SeqA binds with high specificity to hemi-methylated DNA, as formed transiently during replication (Molina and Skarstad, 2004; Waldminghaus and Skarstad, 2009).

**Fig. 2:**
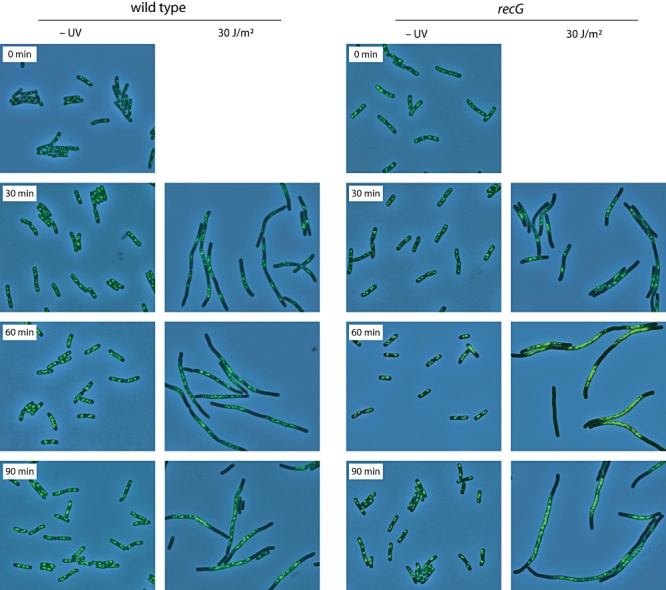
UV-irradiation increases the number of SeqA foci in replicating cells. Strains for eCFP–SeqA expression carried plasmid pDIM083. Expression was induced by adding arabinose 30 min before UV-irradiation and continued throughout the duration of the experiment. Combined phase contrast and fluorescence images are shown. As overexpression has been shown to cause segregation defects (Waldminghaus and Skarstad, 2009), a mock-irradiated control was run in parallel, showing that the effects observed are caused by UV-irradiation rather than SeqA overexpression. The strains used were MG1655 (wild type) and N4560 (*recG*).

Figure 2 demonstrates that some four to six SeqA foci are detected on average in unirradiated wild-type and *recG* cells during exponential growth at 37°C. This fits well with the expected number of replication forks given that cells at this stage of growth have some two to three copies of the origin region on average and one copy of the terminus (Rudolph *et al.*, 2009). The pattern changed drastically following irradiation. The distinct pattern of foci characteristic of exponential cells is replaced with a cloud of multiple foci formed around the mid cell position as division ceases and the cells grow into longer and longer filaments (Fig. 2). Sixty minutes after irradiation the SeqA foci have spread along the filaments. The amplification of SeqA foci seems somewhat greater in *recG* cells and shows no sign of abating as it does after 90 min in wild-type cells. This evidence of persistent and elevated replication may contribute to the very pronounced delay in division of irradiated *recG* cells (Rudolph *et al.*, 2009). These data suggest that the high levels of SDR detected in samples of irradiated *recG dnaA46* cells may coincide with a large increase in hemi-methylated DNA targets for SeqA to bind, which we interpret as evidence of a large increase in the number of replication forks within each cell.

**Fig. 3:**
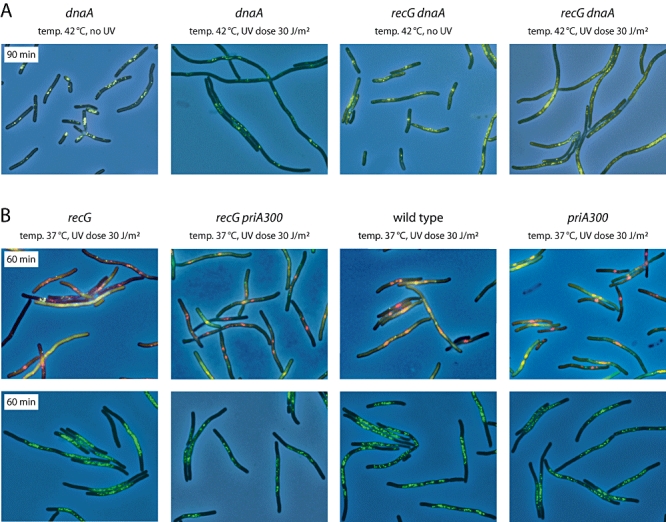
Effect of *oriC* firing and PriA helicase activity on the amplification of SeqA foci. A. Effect of *oriC* firing. The strains used carried the eCFP–SeqA expression construct pDIM083 and were grown to exponential phase at 30°C before samples were irradiated or mock-irradiated with UV and then incubated at 42°C to prevent further firing of *oriC*. Samples were removed and SeqA induced by adding arabinose and incubating for a further 30 min. Combined phase contrast and fluorescence images are shown. The strains used were AU1054 (*dnaA*), AU1091 (*recG dnaA46*). B. Effect of PriA helicase activity. The strains in the upper row carried *lac* and *tet* operator arrays to mark the origin and terminus areas of the chromosome respectively, and pLAU53 to decorate these arrays with fluorescent repressors when required, generating fluorescent foci coloured red (origin) and green (terminus). The strains were RCe72 (*recG*), RCe109 (*recG priA300*), APS345 (wild type) and RCe108 (*priA300*). All four images are reproduced for comparison from Rudolph *et al.* (2009) with permission. The strains in the lower row carried the eCFP–SeqA expression construct pDIM083. Expression was induced by adding arabinose 30 min before UV-irradiation and continued throughout the duration of the experiment. Combined phase contrast and fluorescence images are shown. The strains used were N4560 (*recG*), RCe29 (*recG priA300*), MG1655 (wild type) and N5500 (*priA300*).

Given that *oriC* is known to continue firing at the normal rate after irradiation (Rudolph *et al.*, 2007), at least some of the increase in fork numbers seen in the irradiated *dnaA*^+^ cells may be due to such new initiations. In line with that possibility, we see less amplification of SeqA foci in irradiated *dnaA46* cells at 42°C (Fig. 3A). However, in this case it is very clear that there is a substantially greater amplification of SeqA foci in irradiated *recG dnaA46* cells. This observation indicates that *oriC* firing is not the only factor contributing to the amplification of SeqA foci seen in *dnaA*^+^ cells, consistent with the idea that the induction of SDR establishes new forks elsewhere. However, we note that although there are more SeqA foci in unirradiated *dnaA recG* cells as compared with *dnaA* (*recG*^+^) cells, the difference is not as great as might be expected from the data in Fig. 1. Some of the SeqA foci observed in the *dnaA* single mutant might reflect protein aggregates accumulated in the absence of active replication. Those observed in the irradiated *dnaA* cells are distributed along the filaments, and increased in number, consistent with the increased BrdU labelling detected (Fig. 1B).

But, the data in Fig. 3A do not exclude the possibility that SDR itself is an artefact due to the absence of *oriC* firing. Indeed, all previous studies described SDR in cells in which *oriC* firing is prevented (Kogoma, 1997). Given that replisome components are limited to around 10 DNA polymerase III holoenzymes per cell (Kelman and O'Donnell, 1995), the absence of *oriC* firing might allow their recruitment by PriA instead, enabling synthesis to initiate at other sites. To investigate this possibility, we examined SeqA foci in *dnaA*^+^ cells in which the helicase activity of PriA was eliminated. The increase in the number of foci following irradiation should then be reduced as the absence of PriA helicase activity limits SDR (Tanaka *et al.*, 2003; Rudolph *et al.*, 2009). This is what we observed. The number and intensity of SeqA foci are clearly reduced in both *priA300* and *recG priA300* cells compared with *priA*^+^ controls, while the pattern of origin and terminus replication appears largely the same at this time point (Fig. 3B). However, the reduction is not large and it is noticeable that the number of SeqA foci remains significantly higher than in unirradiated cells. This can be explained by continued *oriC* firing and also the fact that *priA300* does not eliminate SDR entirely. Much of the SDR induced by UV light has been attributed to PriA-mediated replisome assembly at D-loops generated by RecBCD- and RecA-mediated initiation of recombination (Kogoma, 1997), which may proceed to some extent without the need for PriA helicase activity (Zavitz and Marians, 1992). Thus, taken together, our data indicate that SDR occurs in *dnaA*^+^ cells and is not just a pathological consequence of the absence of origin firing, and we conclude that its induction does indeed lead to an increase in the number of forks traversing the chromosome.

### Pathological replication is largely independent of the UV dose

It is very noticeable from the data in Figs 1 and 2 that the replication induced by UV light continues unabated long after the 30–40 min needed to remove the vast majority of pyrimidine dimers (Rudolph *et al.*, 2009). Therefore, we considered the possibility that DNA damage is only the initial trigger for the formation of additional replication forks and that the perpetuation of SDR is the result of secondary replication forks initiated as a result of pathological events arising from unscheduled collisions between opposing forks (Hiasa and Marians, 1994; Krabbe *et al.*, 1997; Markovitz, 2005). Furthermore, we considered the possibility that RecG normally limits this secondary pathology, hence explaining the much-elevated SDR and much-prolonged defects in chromosome segregation and cell division (Rudolph *et al.*, 2009). If true, SDR should be able to continue over extensive periods in *recG* cells almost regardless of the applied UV dose, provided that enough lesions were introduced to trigger the initial events.

**Fig. 4:**
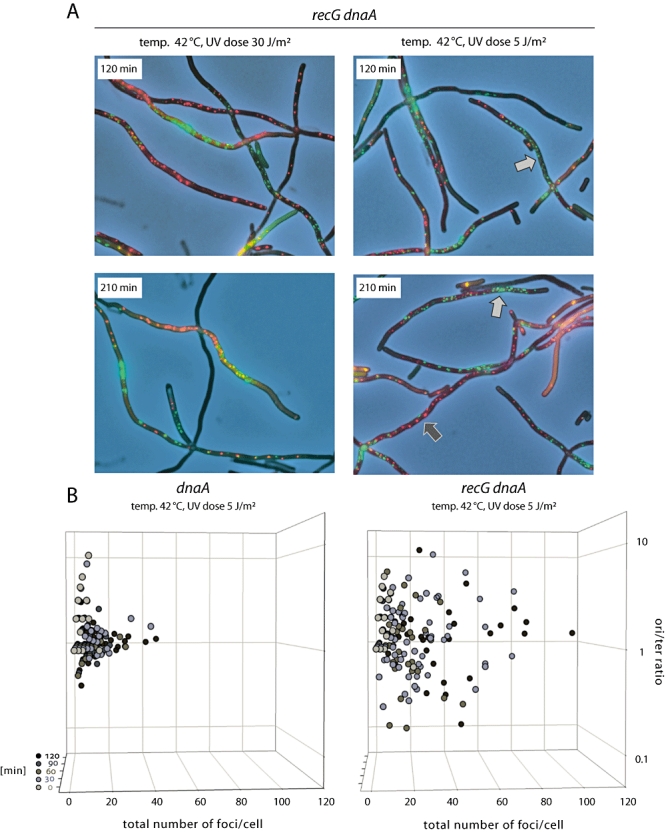
UV-induced SDR leads to dose-independent uncontrolled DNA amplification. A. UV-irradiation leads to a drastic increase in origin (red) and terminus (green) foci in the absence of *oriC* firing (combined phase contrast and fluorescence images are shown), which appears to be largely dose-independent. The light arrows point to filaments with the number of termini exceeding the number of origins whereas the dark arrow indicates a filament with the number of origins exceeding the number of termini. Cells were grown at permissive temperature prior to UV treatment and shifted to 42°C directly after irradiation. The strain used was RCe198 (*recG dnaA46*). Data for *recG dnaA46* irradiated with 30 J m^−2^ are reproduced from Rudolph *et al.* (2009) with permission. B. Analysis of the origin to terminus ratio in UV-irradiated filaments in the absence of *oriC* firing. At every time point indicated the numbers of origin and terminus foci per filament were counted for at least two microscopic fields coming from two independent experiments. The origin/terminus ratio of individual cells was plotted against the total number of foci (origins + termini). The time points are distinguished by different shades of grey as indicated. The data are from two independent experiments, with evaluation of at least one microscopic field per experiment. The experiments for *recG dnaA46* were done using two different *recG* null alleles that gave identical results (RCe198 and RCe246). The experiments for the *dnaA46* single mutant utilized RCe197.

To investigate this possibility, we examined chromosome replication using strains in which origin and terminus areas of the chromosome were tagged with *lac* and *tet* operator arrays respectively, and in which we induced fluorescent repressors in samples of these cells to decorate the arrays. We also used *dnaA46* to prevent *oriC* firing and examined the numbers of origin and terminus foci induced at two different UV doses. We observed that reducing the UV dose sixfold made little difference. The amplification of both origin and terminus areas of the chromosome was as extreme as with the higher dose (Fig. 4A). This explains why very low levels of UV damage are able to induce a very extensive and persistent filamentation phenotype in the absence of RecG (Ishioka *et al.*, 1997; Rudolph *et al.*, 2009). This observation supports the idea that the persistence of SDR is largely a consequence of some secondary event.

Some of the very extensive filaments formed by the *recG dnaA46* cells appeared to show a greatly increased number of terminus foci over origin foci (Fig. 4A, white arrow). We therefore estimated the number of both origin and terminus foci per filament in both *dnaA46* and *recG dnaA46* cells in order to see if there were differences between the two cell types that provided further insight into the function of RecG. Although the clustering of foci made it difficult to obtain accurate counts, the estimates obtained provided clear enough evidence that there was an increase of both origin and terminus foci in *dnaA46* cells 30 min following UV-irradiation (Fig. S2A and B). This was followed by a decrease 60 min later when filaments with a low number of origin foci dominated the culture. This reduction fits with our observation that the majority of wild-type cells show a single cell division following UV-irradiation (Rudolph *et al.*, 2007). One hundred and twenty minutes following irradiation, the number of foci per filament increased again and established filaments with high numbers of both origin and termini foci. Plotting the origin to terminus ratio for each time point in relation to the total number of origin and terminus foci per filament revealed a mild overrepresentation of origin over terminus foci in a small number of the *dnaA46* cells and a very mild overrepresentation of the terminus over the origin in an even smaller fraction of these cells (Fig. 3B).

The situation proved quite different in *recG dnaA46* cells. UV-irradiation led to an almost constant accumulation of origin and terminus foci per filament (Fig. S2C and D). The *ori/ter* ratio fluctuated far more dramatically than with *dnaA46* cells, with some filaments showing a more than sixfold overrepresentation of origin over terminus foci (e.g. 43 *ori*/7 *ter* foci), while others showed a greatly increased number of termini foci (e.g. 5 *ori*/44 *ter* foci) (Fig. 3B). These data indicate that amplification of one chromosomal region does not necessarily lead to duplication of the entire chromosome (Rudolph *et al.*, 2009). We conclude that damage-induced SDR is far more pathological in the absence of RecG and can lead to rapid and uncontrolled over-replication of limited chromosomal areas.

### RecG reduces accumulation of branched DNA intermediates

**Fig. 5:**
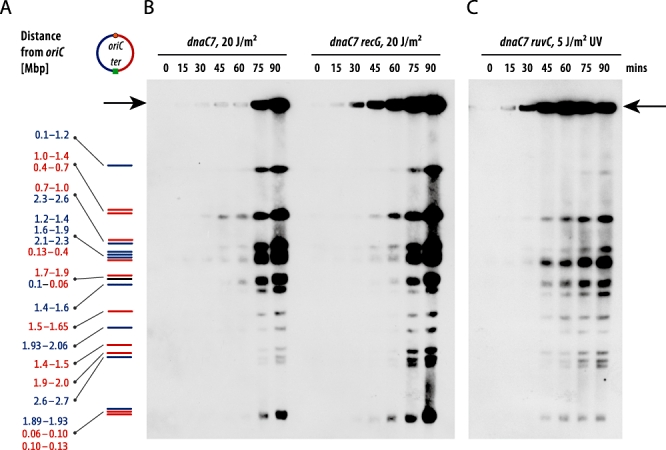
Accumulation of branched DNA after irradiation with UV light. A. Schematic NotI restriction pattern of the *E. coli* chromosome. The distance from *oriC* to each end of the fragments is indicated. Fragments clockwise and anticlockwise of *oriC* are shown in red and blue respectively; the fragment containing *oriC* is shown in black. B and C. Accumulation of non-migrating DNA (black arrow) in synchronized and irradiated cells of RCe79 (*dnaC7*), RCe111 (*dnaC7 recG*) and RCe113 (*dnaC7 ruvC*) after shifting to permissive temperature.

To investigate whether the high level of SDR induced in the absence of RecG is associated with the formation of multiply branched replication intermediates, we used the thermosensitive *dnaC7* allele to synchronize cells at 42°C before shifting to 30°C to allow initiation of replication and adding BrdU to label the newly replicated DNA. Note that *dnaC7* blocks all replication at 42°C, whereas SDR is by definition independent of DnaA. Samples of the cells were taken at intervals, chromosomal DNA extracted in agarose plugs, digested with NotI and the fragments separated via PFGE before probing for BrdU (Fig. 5B) (Rudolph *et al.*, 2007; 2009). Under these conditions, incorporation of label in unirradiated cells is detected fairly soon after the shift to 30°C, whether these cells are *recG*^+^ or *recG*^–^ (Fig. S3B). However, incorporation is delayed following UV-irradiation, consistent with forks stalling as they encounter lesions (Rudolph *et al.*, 2007; 2009). This UV-induced delay is similar in relative terms to that seen with unsynchronized cells (Fig. S1C and D). Labelling resumes as stalled forks are rescued and DNA lesions are excised, enabling replication to proceed without much further hindrance (Rudolph *et al.*, 2007).

A substantial fraction of the label detected under these conditions appears in DNA that fails to migrate from the well. This species is more pronounced following irradiation and, more significantly, appears earlier and accumulates to a higher level in the absence of RecG (Fig. 5B). Its early appearance is particularly noticeable given UV induces a general delay in DNA synthesis. Non-migrating DNA forms an even larger fraction of the labelled species in irradiated cells lacking RuvABC at early time points, despite the lower UV dose used to compensate for the reduced survival of UV-irradiated *ruv* cells (Fig. 5C), suggesting that some might be comprised of NotI DNA fragments linked via Holliday junctions.

**Fig. 6:**
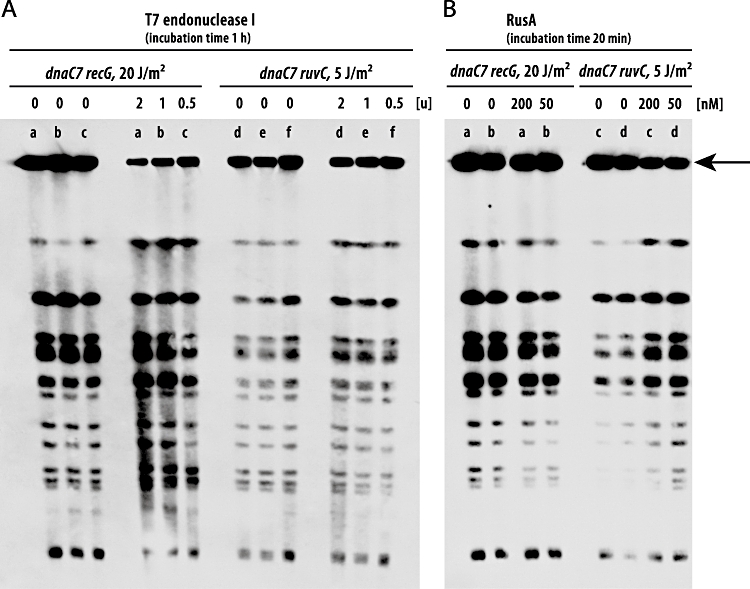
Susceptibility of UV-induced non-migrating DNA structures to T7 endonuclease I or RusA. A and B. Cells were UV-irradiated after synchronization, and shifted to permissive temperature for 90 min in order to allow accumulation of high-molecular-weight DNA. Chromosomal DNA was prepared in agarose plugs, which were cut in half with one half being treated with either T7 endonuclease I or RusA, as indicated, and the other half left untreated, before separating chromosomal fragments by electrophoresis and probing the DNA for BrdU. The labels a–f (A) and a–d (B) indicate the corresponding plug halves. The strains used were RCe111 (*dnaC7 recG*) and RCe113 (*dnaC7 ruvC*).

To identify the DNA species retained in the well, we subjected the NotI-digested DNA to various nucleases prior to electrophoretic analysis. No high-molecular-weight material is observed following additional treatment with a restriction endonuclease that has a high number (∼700) of target sites in the *E. coli* chromosome (Fig. S3D), confirming that the material is susceptible to nuclease digestion. However, the material accumulated in *recG* and *ruvC* cells show differences in susceptibility to nucleases that target branched DNA. The majority of the material accumulated in *recG* cells is eliminated by treatment with T7 endonuclease I (Fig. 6A), consistent with the presence of branched DNA structures. But very little is removed by a Holliday junction resolvase (RusA) (Fig. 6B) that targets Holliday junctions with high specificity and efficiency (Bolt and Lloyd, 2002).

The non-migrating material accumulated in *ruvC* cells appears more resistant to T7 endonuclease I (Fig. 6A). It can be eliminated, but needs longer incubation periods for the digestion (Fig. S4B, lane e). More significantly, a fraction is susceptible to digestion by RusA, consistent with the expected and reported accumulation of Holliday junction intermediates (Donaldson *et al.*, 2006). This is evident from the reduction in the amount of material retained in the well and the corresponding increase in the intensity of the migrating NotI fragments (Fig. 6B). Although not dramatic, this susceptibility to RusA proved highly reproducible. These data demonstrate that the majority of the high-molecular-weight DNA accumulated in *recG* cells contains branched DNA molecules that include few Holliday junction intermediates, consistent with the presence of RuvABC.

## Discussion

The persistence of unresolved recombination intermediates has been offered previously as an explanation for the defective chromosome segregation and extensive cell filamentation phenotype observed in cells lacking RecG following their irradiation with mild UV doses (Ishioka *et al.*, 1997). This interpretation reflected the reported redundancy between RecG and the RuvABC Holliday junction resolvase (Lloyd, 1991). However, during recent studies revisiting the *recG* mutant phenotype, several observations led us to question whether unresolved Holliday junctions were a major factor. We provided evidence that UV-irradiated *recG* cells show drastically increased levels of DnaA-independent DNA synthesis and demonstrated that inactivation of the helicase activity of PriA led to a robust suppression of the UV-induced cell filamentation and a massive reduction in DnaA-independent synthesis. These and other observations opened the possibility that the phenotype of *recG* mutants is due mainly to extensive PriA-mediated over-replication of the chromosome.

In this study we demonstrate that the extensive DnaA-independent SDR observed in cells lacking RecG can lead to replication of any area of the chromosome without preference. It is further elevated following UV-irradiation, but independently of the applied dose, and persists long after UV lesions have been removed, indicating that once induced, SDR is perpetuated by secondary events that are independent of these initial lesions. This pathological DNA synthesis appears to be associated with an increase in the number of replication forks traversing the chromosome, sometimes with extensive regional amplification of chromosomal sections, and generally with the accumulation of high-molecular-weight branched DNA intermediates that contain few Holliday junctions. Taken together, the data presented fit with a model in which the increased incidence of replication fork collisions arising as a consequence of damage-induced SDR leads to a pathological cascade in the absence of RecG, a cascade that impedes normal chromosomal replication and segregation, leading to the persistent cell filamentation.

SDR differs from normal vegetative replication only in its mode of initiation and ability to allow fork movement towards *oriC*, counter to the direction of normal fork movement (Kogoma, 1997). The number of polymerase III holoenzymes is limited to about 10 per cell (Kelman and O'Donnell, 1995), which would tend to place an upper limit on the number of additional replication forks that could be established under SDR-inducing conditions. However, cell growth continues under these conditions and we cannot rule out the possibility that additional replisome components may accumulate. Indeed, this would be consistent with the cloud of SeqA foci observed in UV-irradiated wild-type and *recG* cells (Fig. 2). This observation, together with the fact that inactivation of PriA helicase specifically reduces SeqA foci formation (Fig. 3), indicates that SDR occurs in cells in which the *oriC*-dependent initiation system is fully functional. Previous studies of SDR exploited only cells in which *oriC* firing is prevented (Kogoma, 1997).

**Fig. 7:**
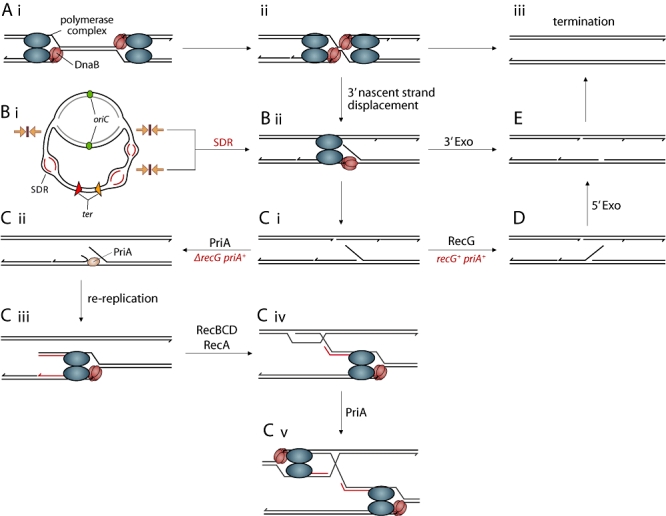
Models depicting possible outcomes of replication fork collision. A. Fork merging and nascent strand ligation. B. Pathological replication resulting from unscheduled replication fork collisions or during normal termination. B(i) Schematic of the *E. coli* chromosome showing normal replication from *oriC* and the presence of several additional replication forks initiated as a result of SDR induction. The opposed arrowheads indicate the positions of unscheduled fork collisions outside of the normal termination zone bounded by Tus*-ter* (for simplicity, only two *ter* sites are depicted). B(ii) Nascent strand displacement following unscheduled collisions triggered by SDR, or at a lower frequency in the absence of SDR. C. Pathological, PriA helicase-dependent replication in the absence of RecG generates a dsDNA branch that can provoke recombination. D. Termination achieved via a 5′ ssDNA exonuclease after RecG converts a 3′ flap to a 5′ flap. E. Termination achieved via a 3′ ssDNA exonuclease.

Our results indicate that following induction of SDR there are many additional replication forks traversing the chromosome and that these forks can extend synthesis to any region of the chromosome, even in the absence of *oriC* firing. The level of SDR is increased in *recG* cells, but it is not immediately obvious why the absence of RecG should have such a catastrophic effect on chromosome segregation and cell division as that reported by Rudolph *et al.* (2009), given that extra forks are also induced in wild-type cells. During normal replication, forks meet in the terminus area and although the details of how termination is achieved remain obscure, it is generally assumed that as the two forks merge and the replisomes dissociate, any remaining gaps in the DNA are filled and the nascent strands sealed by ligase (Fig. 7Ai–iii). The additional replication forks established during induction of SDR would be expected to lead to additional fork collisions, some of which will take place outside of the normal termination zone (Fig. 7Bi). The known ability of RecG to unwind the D-loop and R-loop structures PriA exploits to initiate SDR may explain the much-increased SDR evident in *recG* cells (Vincent *et al.*, 1996; Fukuoh *et al.*, 1997; Kogoma, 1997; McGlynn *et al.*, 1997). However, unless an increased number of collisions of forks can lead to further pathology RecG can limit, it would not be obvious why this increase in the number of forks traversing the chromosome should have such severe consequences for *recG* cells.

Studies of DNA replication termination *in vitro* revealed that without Tus to curb fork movement, the replisome of one fork may displace the 3′ end of the nascent leading strand of the fork coming in the other direction (Hiasa and Marians, 1994). The generation of such a 3′ flap *in vivo* (Fig. 7Bii) may provide a substrate that PriA can then exploit with the aid of its helicase activity to load DnaB and initiate re-replication of the DNA(Fig. 7Ci–iii), which may explain the over-replication seen with *tus* mutants *in vivo* (Krabbe *et al.*, 1997; Markovitz, 2005). In doing so it would generate DNA substrates with duplex DNA ends that provoke RecBCD- and RecA-mediated recombination and thus establish yet new forks (Fig. 7Civ and v). Such a sequence of events would account for the perpetuation of SDR long after the UV lesions inducing SDR had been removed (Fig. 1), and explain the dependence of SDR on RecBCD and RecA (Kogoma, 1997).

RecG has a particularly high affinity for a 3′ flap (McGlynn and Lloyd, 2001; Tanaka and Masai, 2006), and in wild-type cells may limit PriA-mediated re-replication by unwinding the structure and converting it to a 5′ flap that could be subsequently eliminated by a 5′-3′ ssDNA exonuclease (Fig. 7D). In cells lacking RecG, the initial level of SDR would be elevated, leading to even higher levels of unscheduled fork collisions and therefore of 3′ flaps. Without RecG to unwind them, these flaps would have a longer half-life, increasing the opportunity for their targeting by PriA. Furthermore, without RecG, any D-loops established by subsequent recombination would be stabilized, further increasing the likelihood of perpetuating cycles of fork collisions and re-replication. In the meantime, RuvABC would resolve any Holliday junctions generated during the exchanges. This pathological cascade would explain the accumulation of branched DNA intermediates containing few Holliday junctions in irradiated *recG* cells (Figs 5 and 6).

The model outlined in Fig. 7 would predict that 3′-5′ exonucleases might be rather vital in the absence of RecG as they might provide the only means to avoid 3′ flaps from being targeted by PriA, enabling replication to terminate normally (Fig. 7E). In recent studies we have shown that the viability of *recG* cells does indeed depend on the presence of ExoI, ExoVII or the SbcCD nuclease, all of which can digest ssDNA from 3′ ends. We have also shown that this requirement can be overcome by eliminating the helicase activity of PriA (C.J. Rudolph, A.L. Upton and R.G. Lloyd, in preparation).

The model is also in good agreement with data by Donaldson and coworkers, showing that branched DNA structures thought to reflect Holliday junctions can be detected in UV-irradiated wild-type, *ruv* and *recG* cells in a plasmid-based assay (Donaldson *et al.*, 2006). They reported that while the intermediates observed in *ruv* cells persisted, those detected in *recG* cells disappeared as in wild-type cells, as our model would predict, which indicates that they are not the same intermediates that we see accumulating in *recG* cells (Fig. 5). Recent work by Wardrope and coworkers support the conclusion that Holliday junctions do not accumulate in *recG* cells (Wardrope *et al.*, 2009). We assume that some of the high-molecular-weight intermediates we detect are the result of repeated recombinational exchanges, some of which may involve the over-replicated DNA invading the sister chromosome. Thus Holliday junction resolution by RuvABC may lead to the formation of chromosomal multimers (Fig. 7Civ and v). Further repetitions might lead to a network of multiple chromosomal copies tied together, hence the persistence of high-molecular-weight branched DNA species in our study. This interpretation is consistent with the observed synergistic phenotype of *recG* with *xerC* (Meddows *et al.*, 2004). The XerC protein is required for the site-specific recombination at *dif* needed to convert chromosome dimers and higher multimers to monomers (Lesterlin *et al.*, 2004). The equivalent site-specific recombination system of *Bacillus subtilis* is required in order to avoid chromosomal instability following elimination of the Tus analogue, RTP (Lemon *et al.*, 2001), indicating that uncontrolled termination of replication in this species has similar pathological consequences.

The scenario of a replication catastrophe fits well with our observation that UV doses as low as 1 J m^−2^ suffice to induce substantial delays in cell division and that late expression of RecG can efficiently stop the extended filamentation (Ishioka *et al.*, 1997; Rudolph *et al.*, 2009). These observations together with the fact that UV doses differing by sixfold nevertheless lead to similar levels of aberrant chromosome amplification (Fig. 4A) fit well with our model as it predicts that much of the observed pathology is triggered when a fork collision triggers formation of a 3′ flap that PriA subsequently targets. Once triggered, the re-replication of the chromosome perpetuates the problem even when the initiating lesions are long gone. This explains why the expression of *recG* should be able to alleviate the problem at almost any time. First, it would reduce the amount of SDR (Hong *et al.*, 1995; Rudolph *et al.*, 2009). Second, it would help eliminate both the 3′ flap and D-loop intermediates that trigger and perpetuate the cascade, enabling ongoing replication forks to finish multiplication of the chromosomes present, allowing subsequent division of filaments to normal viable cells, as observed (Rudolph *et al.*, 2009).

The idea of a replication catastrophe also predicts that some areas of the chromosome may be re-replicated several times over if formation of multiple forks happens to be induced in close proximity. The cycle of 3′ flap formation, over-replication and formation of additional forks via recombination would then send forks repeatedly through the same area. Such events may account for the frequent asymmetric amplification of the origin and terminus areas of the chromosome apparent in the absence of RecG, which can be quite extreme (Fig. 4). An alternative explanation might be the repeated firing of distinct damage-inducible origins, as previously described (Kogoma, 1997). However, we found no strong evidence of such origins in our analysis of DNA synthesis mediated via SDR (Fig. 1), although we cannot dismiss the existence of such origins. Furthermore, over-initiation at *oriC* has been shown to result in replication fork collapse, as secondary forks are capable of catching up with primary forks, causing the formation of linear DNA fragments due to replication run-off (Simmons *et al.*, 2004). The same rationale should apply to rapidly firing SDR origins, thereby limiting the ability of a single origin to cause over-replication. Finally, we have shown previously that UV-induced synthesis continues after all detectable lesions have been removed (Rudolph *et al.*, 2009). It is not obvious why firing of specific SDR origins should occur once the initial trigger is gone.

Although the data presented here as well as in our previous study are in line with the idea that pathological replication, rather than an accumulation of recombination intermediates, is primarily responsible for the phenotype of cells lacking RecG, they do not rule out the idea that RecG can also help to resolve Holliday junctions, as initially suggested (Lloyd, 1991). However, they raise the possibility that any recombination reaction that increases the number of replication fork collisions may have pathological consequences that reduce viability and hence the ability to recover recombinant products.

The severity of the effects observed begs the question of how replication is brought to an end in eukaryotic cells. It follows from the multiple origins per chromosome that a high frequency of replication fork collisions is the norm, but there is no evidence of the pathology described here. The replicative helicase appears to have the opposite polarity to *E. coli* DnaB (Tuteja and Tuteja, 2004; Costa and Onesti, 2008; Sakakibara *et al.*, 2009). However, it is not clear whether it encircles the (single stranded) leading strand template or whether it encircles double-stranded DNA (Sakakibara *et al.*, 2009). In the latter case, the problem might never arise. However, even in the case that only the leading strand template is encircled, any strand displacement when forks merge would involve the nascent lagging strand and create a 5′ flap rather than a recombinogenic 3′ flap, and would likely be processed via the flap endonuclease, FEN-1. Alternatively, given that Okazaki fragments in eukaryotes are short, the helicase might simply unwind one or more un-ligated fragments completely, leaving no flap. Thus, the difference in polarity may avoid the pathological replication we suggest is a significant risk in bacteria.

## Experimental procedures

### Bacterial strains and plasmids

All strains are derivatives of *E. coli* K-12 (Table 1). For fluorescence microscopy, strains carrying *lacO240* and *tetO240* arrays were transformed with pLAU53, which encodes arabinose-inducible LacI-eCFP (enhanced cyan fluorescent protein) and TetR-eYFP (enhanced yellow fluorescent protein) (Lau *et al.*, 2003). pDIM083 carries the *seqA* coding sequence amplified with primers 5′seqA-BsrGI (5′-cgcatgtctagattagatagttccgcaaaccttctcaatcaattcc) and 3′seqAXbaI (5′-cgcatgtgtacagtgcaatgaaaacgattgaagttgatgatgaactc), cloned behind the P_*araBAD*_ promoter and the eCFP coding sequence of pLAU17 (Lau *et al.*, 2003) using the BsrGI and XbaI sites, thus creating a eCFP–SeqA fusion.

**Table 1:** *Escherichia coli* K-12 strains.

Strain	Relevant Genotype^a^	Source
General P1 donors		
N3793	Δ*recG263::kan*	Al-Deib *et al.* (1996)
RUC663	*tnaA::*Tn*10 dnaA46*	Tove Atlung
MG1655 and derivatives^b^		
MG1655	F^–^*rph-1*	Bachmann (1996)
AM1655	Δ*recG::apra*	R.G., Lloyd and A.A. Mahdi, unpublished
APS345	*att*Tn*7::lacO240*::*kan zdd/e::tetO240::gen*	Rudolph *et al.* (2007)
AU1054	*tnaA::*Tn*10 dnaA46*	MG1655 × P1.RUC663 to Tc^r^
AU1091	*tnaA::*Tn*10 dnaA46*Δ*recG263::kan*	AU1054 × P1.N3793 to Kan^r^
N4560	Δ*recG265::cat*	Mahdi *et al.* (2006)
N5466	Δ*ruvC::cat*	Mahdi *et al.* (2006)
N5500	*priA300*	Jaktaji and Lloyd (2003)
RCe29	*priA300*Δ*recG265::cat*	Rudolph *et al.* (2009)
RCe72	*att*Tn*7::lacO240::kan zdd/e::tetO240::gen*Δ*recG265::cat*	Rudolph *et al.* (2009)
RCe79	*dnaC7*	Rudolph *et al.* (2007)
RCe108	*priA300 att*Tn*7::lacO240::kan zdd/e::tetO240::gen*	Rudolph *et al.* (2009)
RCe109	*priA300 att*Tn*7::lacO240::kan zdd/e::tetO240::gen*Δ*recG265::cat*	Rudolph *et al.* (2009)
RCe111	*dnaC7*Δ*recG265::cat*	Rudolph *et al.* (2009)
RCe113	*dnaC7*Δ*ruvC::cat*	RCe79 × P1.N5466 to Cm^r^
RCe197	*att*Tn*7::lacO240::kan zdd/e::tetO240::gen tnaA::*Tn*10 dnaA46*	Rudolph *et al.* (2009)
RCe198	*att*Tn*7::lacO240::kan zdd/e::tetO240::gen*Δ*recG265::cat tnaA::*Tn*10 dnaA46*	Rudolph *et al.* (2009)
RCe246	*att*Tn*7::lacO240::kan zdd/e::tetO240::gen tnaA::*Tn*10 dnaA46*Δ*recG::apra*	RCe197 × P1.N6052 to Apra^r^

a The abbreviations *kan, cat, gen* and *apra* refer to insertions conferring resistance to kanamycin (Km^r^), chloramphenicol (Cm^r^), gentamicin (Gen^r^) and apramycin (Apra^r^) respectively. Tn*10* confers resistance to tetracycline (Tc^r^). Strains carrying *dnaA46* or *dnaC7* are temperature sensitive for growth. CGSC: Coli Genetic Stock Center, Yale University.

b Only the relevant additional genotype of the derivatives is shown.

### Media and general methods

LB broth and 56/2 salt media, methods for monitoring cell growth, P1*vir* transduction and determining sensitivity to UV have been cited (McGlynn and Lloyd, 2000).

### Fluorescence microscopy

Fluorescence microscopy was as described (Rudolph *et al.*, 2007). Briefly, cells were grown to an A_650_ of 0.2 in LB broth supplemented with 0.5 mM IPTG and 40 ng ml^−1^ anhydrotetracycline. A 1 ml sample was removed and expression induced in this sample at high levels by adding arabinose to 0.2%. The rest of the cells were pelleted, UV-irradiated on the surface of LB agar and resuspended in the original, but filter-sterilized, supernatant to continue incubation. One-millilitre samples were removed every 30 min and expression induced with arabinose for 30 min. A small sample of cells was transferred to a thin 1% LB agarose layer on microscopic slides and visualized with a BX-52 Olympus microscope equipped with a coolSNAP™HQ camera (Photometrics). eCFP and eYFP foci were visualized using the JP4-CFP-YFP filterset 86002v2 (Chroma). Images were taken and analysed by MetaMorph 6.2 (Universal Imaging) and processed using MetaMorph and Adobe Photoshop CS4.

### 5-Bromo-2′-deoxyuridine (BrdU) labelling

BrdU labelling and detection via immunostaining was essentially as described (Rudolph *et al.*, 2007) with some optimizations. Cells were grown in 56/2 salts supplemented with 0.2% casamino acids and 0.32% glucose to an A_650_ of 0.2 and UV-irradiated as for fluorescence microscopy. Cells were resuspended in the sterile filtered supernatant and the first 2 ml sample removed. 5-Bromo-2′-deoxyuridine (BrdU, Sigma) was added to the rest of the culture to 20 μg ml^−1^. Two-millilitre samples were taken at the intervals indicated, pelleted and resuspended in 85 μl TEE buffer (10 mM Tris HCl, 10 mM EGTA, 100 mM EDTA, pH 8.0), containing 0.1% lauroyl-sarcosine and 0.5% SDS. Eighty-five microlitres of liquid 1% low-melting-point agarose was added and the mixture solidified in a disposable plug former (Bio-Rad) at 4°C. Plugs were treated with 10 mg ml^−1^ lysozyme in 3 ml TEE buffer containing 0.1% lauroylsarcosine and 0.5% SDS for 2 h at 37°C and then at 52°C overnight with 5 mg ml^−1^ proteinase K in 3 ml TEE containing 1% SDS. Plugs were washed in TEE for 30 min at 37°C, treated with 1 mM phenylmethane sulphonyl fluoride (freshly prepared as 100 mM stock solution in methanol) in fresh TEE for 1 h at 37°C and washed twice in fresh TEE for 30 min at 37°C. The plugs were subsequently transferred into 300 μl restriction enzyme buffer and incubated for 30 min at room temperature, the buffer changed and 25 u of NotI (NEB) added. Chromosomal DNA was digested overnight and the fragments separated on a 0.8% agarose gel (Bio-Rad pulse field certified agarose) in 0.5× TBE using a CHEF Mapper PFGE system (Bio-Rad), using the running conditions described (Rudolph *et al.*, 2007). DNA was transferred to a Hybond-N + Membrane (Amersham) by alkaline vacuum transfer and UV cross-linked (120 mJ cm^−2^). Blocking was achieved with TBS Tween (50 mM Tris HCl, 150 mM NaCl, pH 8.0, 0.5% Tween 20) containing milk powder. Due to variations in the background intensities of different membrane batches, the milk powder concentration varied between 1.5% and 5%. After blocking the membrane was incubated for 2 h in the presence of mouse anti-BrdU antibody (Santa Cruz), diluted 1:5000 in TBS Tween. Horseradish peroxidase-conjugated secondary antibody (goat anti-mouse, Bio-Rad) was used at a dilution of 1:10 000 for 1.5 h. The membrane was incubated with ECL Plus Western Blotting Detection Reagents (GE Healthcare) and the signal visualized by exposure to X-Omat UV Plus film (Kodak).

### Visualization of high-molecular-weight DNA in UV-irradiated cells

For visualization of high-molecular-weight DNA, *dnaC7* cells were grown at 30°C to an A_650_ of 0.15 before shifting to 42°C for 60 min to bring replication to a halt before irradiating, adding BrdU and shifting back to 30°C. Preparation of chromosomal DNA in agarose plugs for gel analysis and NotI digestion was essentially as described above. For treatment with additional enzymes, the plugs were transferred to fresh buffer (50 mM Tris HCl, pH 7.9; 100 mM NaCl; 10 mM MgCl_2_, 1 mM DTT) and the required enzyme added as indicated. RusA was purified as cited (Bolt and Lloyd, 2002). T7 endonuclease was obtained from NEB.
