# Supplementary material for: Replication fork collisions cause pathological chromosomal amplification in cells lacking RecG DNA translocase
Source: Mol Microbiol. 2009 Oct 26;74(4):940–55. doi: 10.1111/j.1365-2958.2009.06909.x (PMC2788051; doi:10.1111/j.1365-2958.2009.06909.x)
Supplement: Supplementary file 1 [file mmi0074-0940-SD1.pdf]

Replication fork collisions cause pathological chromosomal amplification in cells lacking RecG DNA translocase – Supporting information

*Christian J. Rudolph, Amy L. Upton and Robert G. Lloyd**

Institute of Genetics, University of Nottingham, Queen's Medical Centre, Nottingham, NG7 2UH, United Kingdom

*for correspondence

E-mail bob.lloyd@nottingham.ac.uk

Tel (+44) 115 823 03 03
Fax (+44) 115 823 03 38

Christian J. Rudolph, Amy L. Upton and Robert G. Lloyd

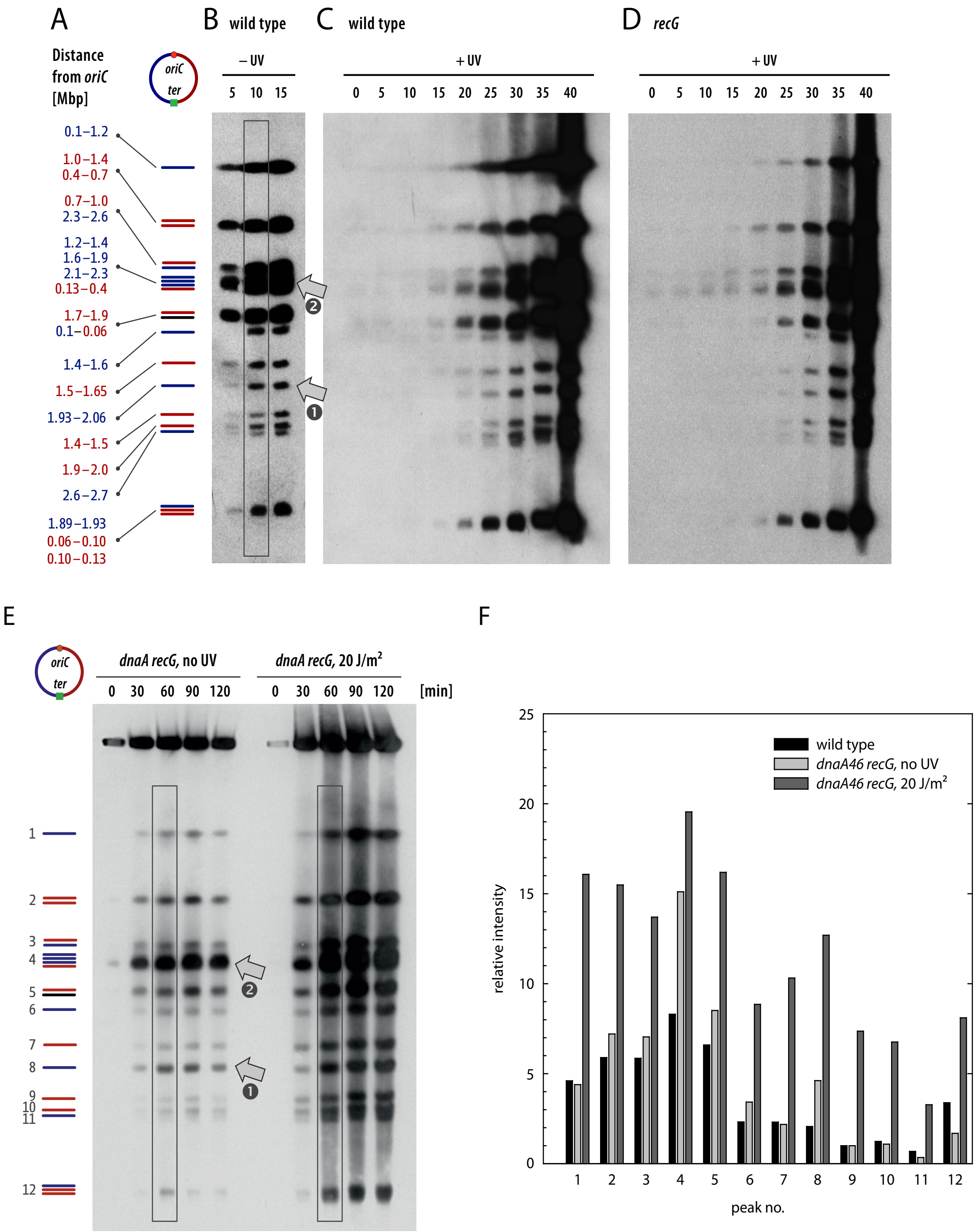

Fig. S1. Replication of UV-irradiated DNA. (A) Schematic *Not*I restriction pattern of the *E. coli* chromosome. The distance from *oriC* to each end of the fragments is indicated. Fragments clockwise and anticlockwise of *oriC* are shown in red and blue, respectively. (B) Fluorograph showing BrdU incorporation into the chromosome of mock-irradiated wild type strain MG1655. The arrows mark two specific fragments which show disproportionate labelling in a *dnaA46 recG* double mutant (compare with panel E and Fig. 1C and 1D) but not in wild type cells. The rectangle indicates the lane used for the analysis of band intensities shown in panel F. (C) Fluorograph showing BrdU incorporation into the chromosome of UV-irradiated wild type strain MG1655. (D) Fluorograph showing BrdU incorporation into the chromosome of UV-irradiated strain N4560 *(recG)*. (E) Visualisation of BrdU incorporation in UV-irradiated or mock irradiated cells in the absence of *oriC* firing (reproduced from Fig 1D for comparison). After growth at permissive temperature, cells were UV irradiated or mock irradiated and subsequently shifted to 42 °C. At the time points indicated the cells were pulse labelled with BrdU for 10 min. The strain used was AU1091 *(recG dnaA46)*. The arrows indicate two fragments that show an increased amount

continues page 3

Christian J. Rudolph, Amy L. Upton and Robert G. Lloyd

Fig. S1 continued

of labelling in comparison to wild type cells (compare with panel B). The rectangle indicates the lane used for the analysis of band intensities shown in panel F. (F) Semi-quantitative analysis of band intensities of the lanes indicated in panels B and E. Quantifiable bands were numbered as shown in panel E. Signal intensities of bands for the unirradiated wild type sample (panel B) were measured relative to band no. 9, which is a clearly defined, non-saturated single fragment band. Band intensities for both the mock- and UV-irradiated *dnaA46 recG* samples were measured relative to the intensity of band no. 9 of the unirradiated *dnaA46 recG* sample, thus allowing some indication of the increase in synthesis caused by SDR following irradiation.

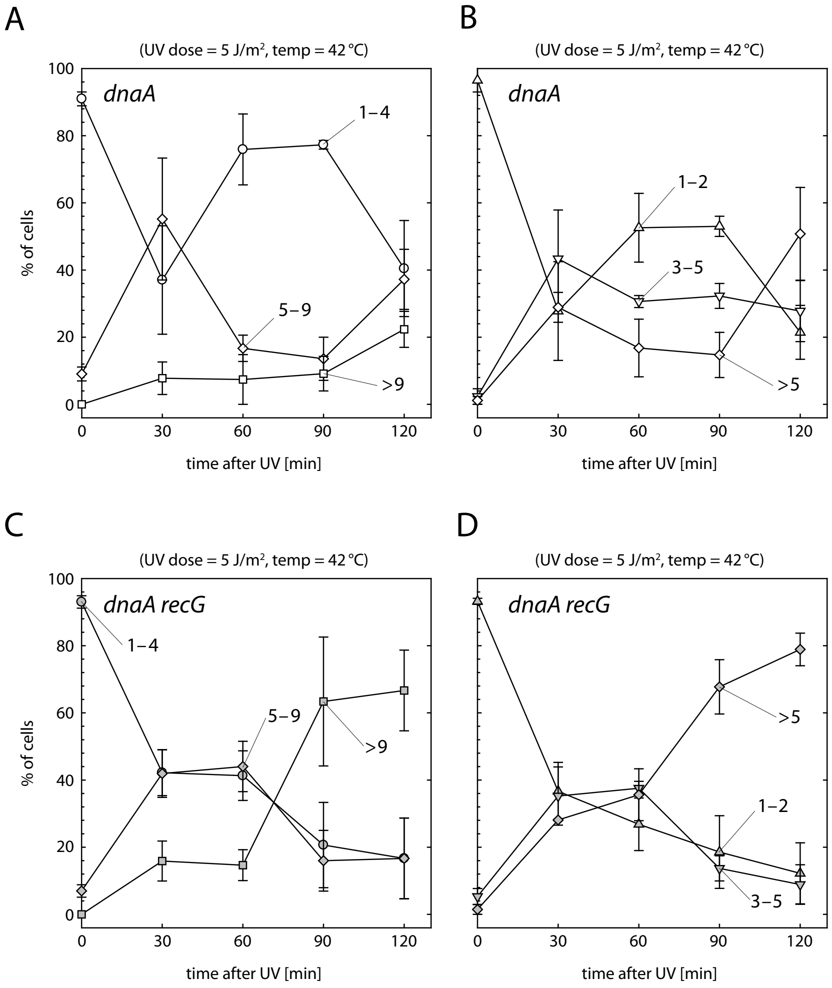

Fig. S2. Effect of UV on multiplication of the origin and terminus area in *dnaA46* and *dnaA46 recG* cells. For quantification of the number of origin (A & C) and terminus (B & D) foci, microscopical fields from two independent experiments were analysed. For every cell the origin and terminus foci were counted. The cells were then divided into classes. For origin foci, 90 % of unirradiated cells were found to contain no more than 4 foci, leading to the definition of a first class of cells with 1–4 foci. Cells with 5–9 foci counted as a class with an elevated number of foci. A third class with 10 or more foci represents filaments with a highly elevated number of foci. Similar classes were defined for the number of termini foci per cell (1–2 [>95 % in unirradiated cells], 3–5 and more than 5 foci per cell). Cells which lacked either the origin or terminus signal or both were not included in the calculation. (A–B) Changes in the number of origin (A) and terminus (B) foci per cell in *dnaA46* cells irradiated with 5 J/m^2 UV and shifted to restrictive temperature. The strain used was RCe197. (C–D) Changes in the number of origin (C) and terminus (D) foci per cell in *dnaA46 recG* cells irradiated with 5 J/m^2 UV with subsequent shift to restrictive temperature. The strain used was RCe198.

Christian J. Rudolph, Amy L. Upton and Robert G. Lloyd

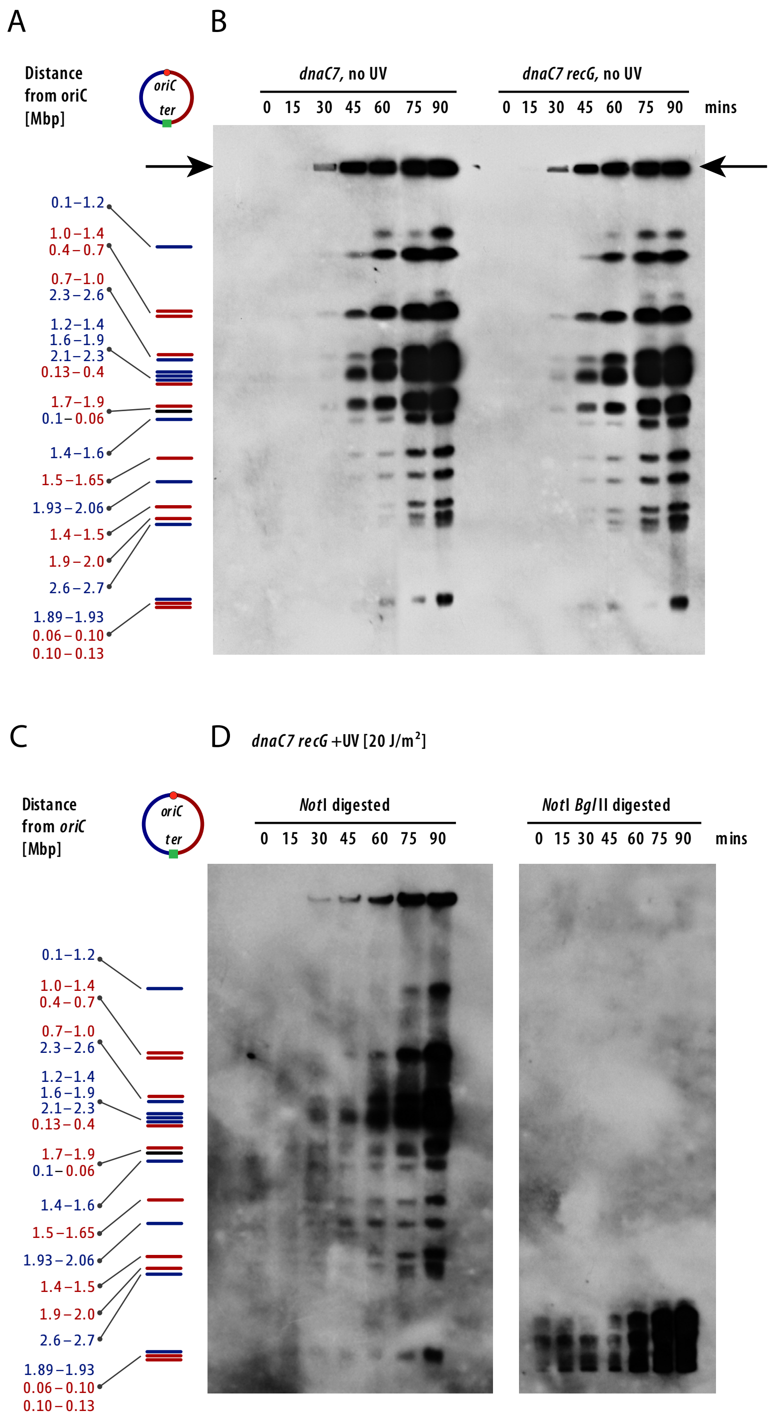

Fig. S3. Accumulation of non-migrating, high molecular weight DNA in *dnaC7* and *dnaC7 recG* cells. (A & C) Schematic *Not*I restriction pattern of the *E. coli* chromosome. The distance from *oriC* to each end of the fragments is indicated. Fragments clockwise and anticlockwise of *oriC* are shown in red and blue, respectively. (B) Accumulation of non-migrating DNA (black arrow) in synchronised *dnaC7* and *dnaC7 recG* cells after shifting to permissive temperature. The strains used were RCe79 *(dnaC7)* and RCe111 *(dnaC7 recG)*. (D) The high molecular weight DNA accumulated in UV irradiated *dnaC7 recG* (RCe111) cells following UV irradiation can be completely digested with *Bgl*II, which cuts the *E. coli* chromosome with high frequency (~700 recognition sites).

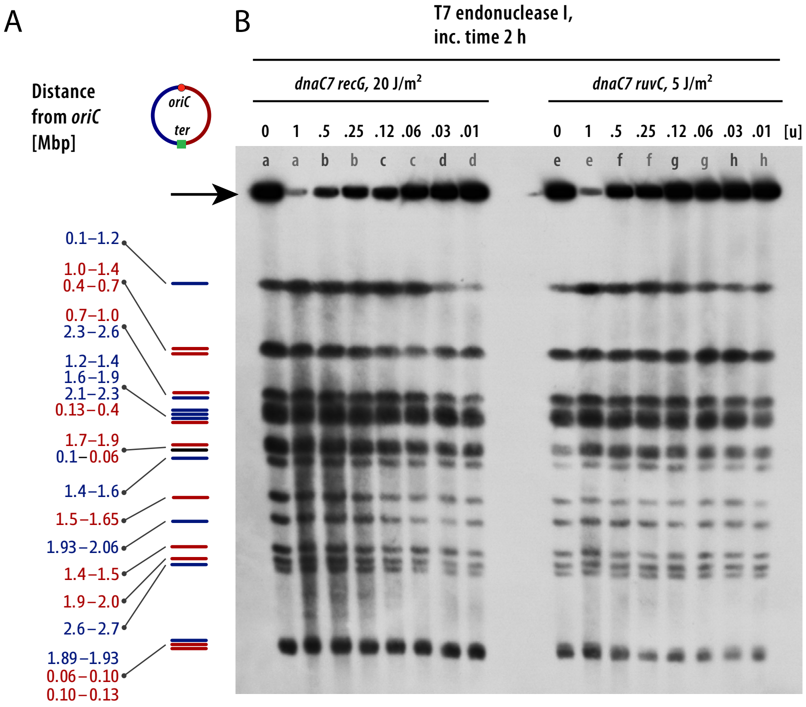

Fig. S4. Susceptibility of branched DNA accumulated following UV irradiation in *dnaC7 recG* and *dnaC7 ruvC* cells to T7 endonuclease I. (A) Schematic *Not*I restriction pattern of the *E. coli* chromosome. The distance from *oriC* to each end of the fragments is indicated. Fragments clockwise and anticlockwise of *oriC* are shown in red and blue, respectively. (B) Susceptibility of the non-migrating DNA to various concentrations of T7 endonuclease I. Chromosomal DNA was prepared in agarose plugs, which were cut in half with one half being treated with T7 endonuclease I, as indicated, and the other half left untreated, before separating chromosomal fragments by electrophoresis and probing the DNA for BrdU. The labels a–h indicate the corresponding plug halves. The strains used were RCe111 *(dnaC7 recG)* and RCe113 *(dnaC7 ruvC)*.
